# Spider Ecology and Behaviour—Spiders as Model Organisms

**DOI:** 10.3390/insects14040330

**Published:** 2023-03-28

**Authors:** Thomas Hesselberg, Dumas Gálvez

**Affiliations:** 1 Department for Continuing Education, University of Oxford, Oxford OX1 2JA, UK; 2 Department of Biology, University of Oxford, Oxford OX1 3PJ, UK; 3 Coiba Scientific Station, Panama City 0843-01853, Panama; 4 Programa Centroamericano de Maestría en Entomología, Universidad de Panamá, Panama City 0824, Panama; 5 Smithsonian Tropical Research Institute, Panama City P.O. Box 0843-03092, Panama

## 1. Introduction

Spiders are versatile and ubiquitous generalist predators that can be found in all terrestrial ecosystems except for Antarctica. Therefore, it is, perhaps, unsurprising that they have been studied fairly extensively within many of the subdisciplines that make up ecology and animal behaviour. In ecology, they are prominently featured in studies, particularly on dispersal and biogeography [1,2], due to their unique ability for long-distance dispersal via ballooning [3], and in studies on niche separation [4,5]. In behavioural studies, they are model organisms for studies in animal communication and signalling [6], foraging behaviour [7], mating behaviour and animal contests [8,9], and cognition [10,11], while web-building spiders, in particular, are also used extensively in studies on construction behaviour and behavioural flexibility [12,13]. More recently, it has also become evident that spiders, in addition to their intrinsic interest as fascinating and, illogically, feared animals, likely due to press misinformation [14], may be of direct benefit to society, a field we can call applied arachnology similar to, or a subset of, the more established field of applied entomology. Areas of particular interest to behaviour and ecology include their role as enemies of natural pests [15,16], their webs as indicators of pollution [17,18], and their significant potential in biomimetics, which is the inspiration, abstraction, and application of evolved processes or traits in biological organisms to our technology [19]. The biomimetics potential of spider behaviour includes biologically inspired locomotion and robotics [20,21], and using the spider web for inspiration for sensors [22] and light weight composite structures [23].

This Special Issue reflects the diverse range of topics within ecology and behaviour that can be fruitfully studied using spiders as model organisms. Below, we give a brief overview of the papers featured in the Special Issue in the context of applied and basic research and highlighting two papers that evaluate and develop new methods for studying their behaviour.

## 2. Applied Arachnology

One of the most well-studied areas of applied arachnology is undoubtedly arachnids’ potential role as natural enemies of agricultural pests. Traditionally, the most focus has been on mites, which can act both as pests [24] and as natural pest controllers [25]. However, the role of spiders as important regulators of pest species in agroecosystems, in combination with parasitic wasps and other insect predators, is becoming more established [16]. In the present Special Issue, Thomas Roberts-McEwen and colleagues [26] show that a group-living araneid, the tropical tent-web spider, *Cyrtophora citricola*, might have hitherto overlooked potential in controlling the tomato leafminer (*Tuta absoluta*), a major pest on tomato plants worldwide. Choice experiments demonstrated that the tent-web spider had nearly similar capture efficiency between tomato leafminers and mutant flight-less *Drosophila*, and had a far higher capture success rate than against the larger black soldier flies. This, combined with observational data from Southern Spain on web sizes in different seasons, suggests that the tent-web spider could potentially be a successful biological control agent of the tomato leafminer in the tomato planting and growing season assuming that high parasitic wasp infection of spider eggs can be controlled [26].

## 3. Basic Research on Spider Ecology and Behaviour

Spider foraging strategies, and thus, to some extent, most aspects of their ecology and behaviour, can be split into either active roaming spiders that do not build a web (cursorial spiders) or sit-and-wait web-building spiders. The Special Issue includes two papers that focus on cursorial spiders, and five papers with a main focus on web-building spiders.

### 3.1. Cursorial Spiders

Maria Trabalon [27] looked at spider breeding welfare and compared the body mass and locomotory and exploratory behaviour of the wolf spider, *Pardosa saltans*, between spiders, immediately after they were caught, wild-caught spiders after being kept 15 days in the laboratory and laboratory-reared spiders. The results showed that while laboratory rearing increased body mass, it reduced behavioural activities, although this reduction could be mitigated by providing litter to the rearing chambers. Marzena Stańska and Tomasz Stański [28] compared assemblages, including both cursorial and web-building spiders inhabiting the optimal, terminal/decay, and regeneration phases of a primeval forest in Poland. Interestingly, the study suggests that the highest species diversity is found in the terminal/decay phase, possibly due to more niches in that phase, while the regeneration phase had the lowest.

### 3.2. Web-Building Spiders

We start this section on web-building spiders with a very interesting group of araneid spiders that have lost the ability to build full orb webs—the bolas spiders, which, instead of a web construct, they use a single thread as a lasso to catch moths attracted by the pheromones emitted by the spider [29]. Candido Dias, Jr and colleagues looked closely at the biomechanics behind this fascinating prey capture behaviour. In the first paper [30], Candido Dias, Jr and John Roff showed, using high-speed cameras, that the South African grassland bolas spider, *Cladomelea akermani*, actively spins, not only the bolas, but also its body. They were able to show with computational fluid dynamics models that this spinning likely has the function of further spreading the emitted pheromones in open habitats. In the second paper [31], Candido Dias, Jr. and John Long, Jr. analysed the prey capture behaviour of the American bolas spider, *Mastophora hutchinsoni*, by calculating the kinematics of both spider and moth based on high-speed recordings to model the physical properties of the bolas during prey capture. Their model showed that the material properties of the glue in the bolas of *M. hutchinsoni* are different to that of previously studied bolas spiders.

The Special Issue also features two brief reports on spiders and invasive species. In the first [32], Arty Schronce and Andrew Davies studied an interesting interaction in the US between the invasive Joro spider, *Trichonephila clavate*, and the native northern cardinal, where the bird perches on top of the very strong orb web and steals prey items directly from the web. In the second [33], El Ellsworth and colleagues looked at how invasive plants and other management strategies impact spider communities (predominantly web-building spiders) in five parks in the greater Memphis area in the US. The study showed that invasive plants can serve as a useful habitat for native spiders as exemplified by the native humpbacked orb-weaver, *Eustala anastera*, being found exclusively on the invasive Chinese privet.

Lastly, in a review, Thomas Hesselberg and colleagues [34] looked more broadly at the associations between web-building spiders and specific host plant species, including a brief overview of cursorial spider-plant associations. The study confirms that associations between spiders and plants are rare, but also found two promising candidates for further studies. The Australian linyphiid *Laetesia raveni* is exclusively reported from two thorny plant species, and two species of Central American araneids in the genus *Eustala* are tightly associated with ant-protected acacia trees.

## 4. New Methodologies for Studying Spider Ecology and Behaviour

Novel methodologies or approaches to conducting research is often a major driver for important research breakthrough, and the field of spider ecology and behaviour is no exception. The field is particularly diverse in its methodology ranging from low-tech, cheap, and simple experimental approaches [35] to high-tech computational or experimental approaches [36]. The Special Issue includes two papers from each of these extremes. In the latter category, Nathan Justus and colleagues [37] developed a clever high-tech integrated system of combining stereo vision and video vibrometry to automatically gather 3D vibrational information to study signal propagation in spider webs, which they successfully validated using laser vibrometry in webs of black widows (*Latrodectus hesperus*). In the former category, Mollie Davies and Thomas Hesselberg [38] reviewed and updated a cheap and easy, old technique of studying behaviour of orb web-building spiders in the field using a tuning fork. They showed that while high-frequency tuning forks (440 Hz) mostly elicited prey capture behaviour in the tetragnathid spider *Metellina segmentata*, a lower-frequency tuning fork (256 Hz) tended to elicit escape behaviour.

## 5. Conclusions

Hopefully, it will be clear from the overview given above that the Special Issue covers a wide range of topical areas of spider research within the fields of ecology and behaviour. We hope that the ten papers included in the issue will contribute to further stimulate research in ecology and behaviour using spiders as model organisms.

## References

[1] Bronte D., Clobert J., Baguette M., Benton T.G., Bullock J.M. (2012). Case-study II—spiders as a model in dispersal ecology and evolution. Dispersal Ecology and Evolution.

[2] Gillespie R.G. (2002). Biogeography of spiders on remote oceanic islands of the Pacific: Archipelagoes as stepping stones?. J. Biogeogr..

[3] Weyman G.S. (1993). A review of the possible causative factors and significance of ballooning in spiders. Ethol. Ecol. Evol..

[4] Brown K.M. (1981). Foraging ecology and niche partitioning in orb-weaving spiders. Oecologia.

[5] Sanders D., Vogel E., Knop E. (2015). Individual and species-specific traits explain niche size and functional role in spiders as generalist predators. J. Anim. Ecol..

[6] Oxford G.S., Gillespie R.G. (1998). Evolution and ecology of spider colouration. Annu. Rev. Entomol..

[7] Uetz G. (1992). Foraging strategies of spiders. Trends Ecol. Evol..

[8] Arnott G., Elwood R.W. (2009). Assessment of fighting ability in animal contests. Anim. Behav..

[9] Elias D.O., Kasumovic M.M., Punzalan D., Andrade M.C.B., Mason A.C. (2008). Assessment during aggressive contests between male jumping spiders. Anim. Behav..

[10] Jackson R.R., Cross F.R., Casas J. (2011). Spider Cognition. Spider Physiology and Behaviour.

[11] Japyassú H.F., Laland K.N. (2017). Extended spider cognition. Anim. Cogn..

[12] Eberhard W.G. (2020). Spider Webs—Behaviour, Function and Evolution.

[13] Hesselberg T. (2015). Exploration behaviour and behavioural flexibility in orb-web spiders: A review. Curr. Zool..

[14] Mammola S., Malumbres-Olarte J., Arabesky V., Barrales-Alcalá D.A., Barrion-Dupo A.L., Benamú M.A., Bird T.L., Bogomolova B.M., Cardoso P., Chatzaki M. (2022). The global spread of misinformation on spiders. Curr. Biol..

[15] Sunderland K. (1999). Mechanisms Underlying the Effects of Spiders on Pest Populations. J. Arachnol..

[16] Michalko R., Pekár S., Entling M.H. (2019). An updated perspective on spiders as generalist predators in biological control. Oecologia.

[17] Samu F., Vollrath F. (1992). Spider orb web as bioassay for pesticide side effects. Entomol. Exp. Appl..

[18] Stojanowska A., Zeynalli F., Wróbel M., Rybak J. (2023). The use of spider webs in the monitoring of air quality—A review. Integr. Environ. Assess. Manag..

[19] Lenau T.A., Metze A.-L., Hesselberg T. Paradigms for biologically inspired design. Proceedings of the SPIE Smart Structures and Materials + Nondestructive Evaluation and Health Monitoring.

[20] Vidoni R., Gasparetto A. (2011). Efficient force distribution and leg posture for a bio-inspired spider robot. Robot. Auton. Syst..

[21] Vollrath F., Krink T. (2020). Spider webs inspiring soft robotics. J. R. Soc. Interface.

[22] Zhou J., Lai J., Menda G., Stafstrom J.A., Miles C.I., Hoy R.H., Miles R.N. (2022). Outsourced hearing in an orb-weaving spider that uses its web as an auditory sensor. Proc. Natl. Acad. Sci. USA.

[23] Regassa Y., Lemu H.G., Sibarizuh B., Rahimeto S. (2021). Studies on the Geometrical Design of Spider Webs for Reinforced Composite Structures. J. Compos. Sci..

[24] Takafuji A., Ozawa A., Nemoto H., Gotoh T. (2000). Spider Mites of Japan: Their Biology and Control. Exp. Appl. Acaraol..

[25] McMurtry J.A., Croft C.A. (1997). Life-styles of phytoseiid mites and their roles in biological control. Annu. Rev..

[26] Roberts-McEwen T.A., Deutsch E.K., Mowery M.A., Grinsted L. (2023). Group-Living Spider *Cyrtophora citricola* as a Potential Novel Biological Control Agent of the Tomato Pest *Tuta absoluta*. Insects.

[27] Trabalon M. (2022). Effects of Wolf Spiders’ Captive Environment on Their Locomotor and Exploratory Behaviours. Insects.

[28] Stańska M., Stański T. (2022). Spider Assemblages of Tree Trunks and Tree Branches in Three Developmental Phases of Primeval Oak–Lime–Hornbeam Forest in the Białowieża National Park. Insects.

[29] Yeargan K.N. (1994). Biology of bolas spiders. Annu. Rev. Entomol..

[30] Diaz C., Roff J. (2022). Mechanics of the Prey Capture Technique of the South African Grassland Bolas Spider, *Cladomelea akermani*. Insects.

[31] Diaz C., Long J.H. (2022). Behavior and Bioadhesives: How Bolas Spiders, *Mastophora hutchinsoni*, Catch Moths. Insects.

[32] Schronce A., Davis A.K. (2022). Novel Observation: Northern Cardinal (*Cardinalis cardinalis*) Perches on an Invasive Jorō Spider (*Trichonephila clavata*) Web and Steals Food. Insects.

[33] Ellsworth E., Li Y., Chari L.D., Kron A., Moyo S. (2022). Tangled in a Web: Management Type and Vegetation Shape the Occurrence of Web-Building Spiders in Protected Areas. Insects.

[34] Hesselberg T., Boyd K.M., Styrsky J.D., Gálvez D. (2023). Host Plant Specificity in Web-Building Spiders. Insects.

[35] Zschokke S., Herberstein M.E. (2005). Laboratory methods for maintaining and studying web-building spiders. J. Arachnol..

[36] Mortimer B., Soler A., Siviour C.R., Zaera R., Vollrath F. (2016). Tuning the instrument: Sonic properties in the spider’s web. J. R. Soc. Interface.

[37] Justus N., Krugner R., Hatton R.L. (2022). Validation of a Novel Stereo Vibrometry Technique for Spiderweb Signal Analysis. Insects.

[38] Davies M.S., Hesselberg T. (2022). The Use of Tuning Forks for Studying Behavioural Responses in Orb Web Spiders. Insects.

